# Neutrophil gelatinase-associated lipocalin (NGAL) is a poor diagnostic marker for sepsis in the ICU – An observational multicentre study

**DOI:** 10.1371/journal.pone.0343752

**Published:** 2026-08-14

**Authors:** Lisa Boström, Sofie Hagström, Jonas Engström, Anders Olof Larsson, Hans Friberg, Maria Lengquist, Attila Frigyesi

**Affiliations:** 1 Department of Clinical Sciences, Lund University, Lund, Sweden; 2 Intensive and Perioperative Care, Skåne University Hospital, Lund, Sweden; 3 Anaesthesia and Intensive Care, Kristianstad Hospital, Kristianstad, Sweden; 4 Department of Clinical Chemistry, Uppsala University Hospital, Uppsala, Sweden; 5 Intensive and Perioperative Care, Skåne University Hospital, Malmö, Sweden; Gifu University School of Medicine Graduate School of Medicine: Gifu Daigaku Igakubu Daigakuin Igakukei Kenkyuka, JAPAN

## Abstract

**Background:**

Sepsis is a major public health challenge, and reliable biomarkers are essential for distinguishing sepsis from other conditions. Neutrophil Gelatinase-Associated Lipocalin (NGAL) has shown promise as a diagnostic marker due to its role in the immune response. This study evaluates plasma NGAL as a diagnostic tool at the time of ICU admission.

**Methods:**

We analysed plasma NGAL and C-reactive protein (CRP) levels in 2950 adult patients admitted to four ICUs between 2015 and 2018. All patients were retrospectively screened for Sepsis-3 criteria at ICU admission. The discriminative performance of NGAL and CRP for sepsis was assessed using receiver operating characteristic (ROC) analysis, with NGAL levels adjusted for chronic kidney disease (CKD) and age. Patients were stratified by renal function.

**Findings:**

Plasma NGAL levels were significantly higher in septic patients (p < 0.001). For the whole cohort, NGAL alone yielded an area under the curve (AUC) of 0.66 (confidence interval (CI) 0.64–0.68), CRP yielded an AUC of 0.71 (CI 0.69–0.73, p < 0.001), and combining NGAL with CRP nominally improved discriminative performance (ΔAUC 0.018, 95% CI 0.011–0.027). Stratified analyses indicated that NGAL, together with CRP, significantly outperformed CRP alone in patients with no kidney injury and those with Acute Kidney Injury (AKI) only. In contrast, differences were not significant in patients with CKD only or CKD and AKI.

**Conclusion:**

In this large cohort, NGAL showed modest discrimination for sepsis, with a nominal improvement when combined with CRP. These findings do not indicate that NGAL meaningfully improves sepsis diagnosis in the ICU.

## Introduction

Sepsis remains a significant public health challenge, and improved diagnostic tools are needed [1,2]. Neutrophil Gelatinase-Associated Lipocalin (NGAL), also known as lipocalin-2, is an intriguing biomarker candidate for diagnosing sepsis. This 198-amino-acid glycoprotein belongs to the lipocalin family and is primarily secreted by immune cells, including neutrophils, macrophages, and dendritic cells [3,4]. NGAL exists in three forms: a 25 kDa monomer, a 45 kDa dimer, and a 135 kDa heterodimer covalently bound to gelatinase [3]. It is widely distributed across various tissues, such as the kidney, heart, lung, bone marrow, liver, and adipose tissue, and is upregulated in response to inflammatory and metabolic disorders [4,5].

The monomeric form is the predominant form secreted by renal tubular epithelial cells, and the dimeric form is the predominant form secreted by neutrophils [6]. The heterodimeric form, bound to matrix metalloproteinase-9 (MMP-9), plays a role in extracellular matrix degradation [7].

NGAL´s potential as an early biomarker for Acute Kidney Injury (AKI) has been extensively documented [4,8–10]. Plasma NGAL levels rise rapidly within two hours of AKI onset, peaking at six hours, offering an earlier indication of kidney injury compared to creatinine [4,11,12]. Since 2023, the FDA has approved NGAL as a biomarker for predicting severe AKI in pediatric patients [10].

Beyond its role in kidney injury, NGAL is pivotal in the body’s defence against bacterial infection by sequestering iron-loaded siderophores, thereby inhibiting bacterial growth [7,13]. This iron-withholding mechanism is crucial to the innate immune response, with NGAL regulating iron-dependent genes [9,14].

The role of NGAL in sepsis-associated AKI has been explored in several studies, highlighting that NGAL levels are elevated in sepsis [15–18]. Notably, research by Mårtensson et al. and Paul et al. indicate that the association between plasma NGAL and sepsis are independent of AKI [19,20]. Despite evidence linking NGAL to inflammation and kidney failure [4,16], studies investigating NGAL as a diagnostic marker for sepsis remain limited, constrained by small sample sizes and diverse clinical settings [19,21–25].

This study aims to evaluate, within a large cohort of critically ill patients, the utility of NGAL as a clinical biomarker for distinguishing sepsis from non-sepsis. We hypothesize that NGAL serves as a diagnostic marker for sepsis in the Intensive Care Unit (ICU). For comparative analysis, we also examine the well-established sepsis marker, C-reactive Protein (CRP) [26,27].

## Materials and methods

### Study design and setting

This retrospective observational study used prospectively collected blood samples from the Swecrit biobank [28]. The biobank comprises blood samples from all patients admitted to the ICUs at four hospitals in southern Sweden between 2015 and 2018: Skåne University Hospital in Lund and Malmö, Helsingborg Hospital, and Kristianstad Hospital. For the present study, we included blood samples from patients who met the predefined inclusion criteria.

### Ethics and consent

Ethical approval for the Swecrit biobank and the present study was obtained from the Regional Ethical Review Board in Lund, Sweden (EPN 2015/267 and 2017/802) before study initiation.

The study used prospectively collected blood samples from ICU patients. In accordance with the ethical approval, patient consent was waived for initial sample collection, and samples were stored in the biobank. An opt-out procedure was implemented: 2–3 months after ICU discharge, surviving patients received written information about the study and could decline participation. If a patient chose to opt out, all data and stored samples for that individual were deleted or destroyed.

No minors were included in this study. The study was conducted in accordance with the Declaration of Helsinki and all relevant local guidelines and regulations.

The study was conducted and reported in accordance with the Standards for Reporting Diagnostic Accuracy Studies (STARD) guidelines [29,30] (see S1 Checklist).

### Participants

This study included all ICU patients ≥18 years of age who had a stay > 24 hours or died within the first 24 hours, provided that eligible blood samples were available in the biobank and baseline creatinine measurements were available.

### Data sources and variables

Clinical data were entered by the treating physician into the PasIva software (used to collect data for the Swedish Intensive Care Registry). These variables include vital signs and other variables needed to calculate the Simplified Acute Physiology Score 3 (SAPS-3) and SOFA scores [31,32]. Laboratory values, microbiological testing and results were automatically extracted using the hospital’s electronic laboratory system. Medical records were reviewed for information on the administration of antibiotics and LMCI criteria in culture-negative patients. For biomarkers that were measured multiple times, the values closest to ICU admission (within 24 hours) were selected. CRP was used for comparison based on its current role as the most commonly used marker for sepsis [26,27].

Sepsis was defined as a Sequential Organ Failure Assessment (SOFA) score ≥2 within ±1 hour of ICU admission in combination with a retrospective classification of infection according to the Sepsis-3 criteria [33]. Criteria for infection were either 1) culture positivity within ±48 hours of ICU admission, or 2) culture negativity and suspected infection (blood culture sampling within 24 hours of ICU admission with concomitant antibiotic administration) and probable infection according to the Linder-Mellhammar Criteria of Infection (LMCI) [34,35].

Infection adjudication was performed by five physicians in anesthesiology and intensive care (three residents and two board-certified specialists). All reviewers followed a structured adjudication protocol based on the LMCI criteria and were trained by the same instructor, who was highly familiar with the protocol. In cases where the classification was uncertain, the case was discussed within the reviewer group until consensus was reached. Interrater variability was not formally assessed.

In contrast to NGAL, routine CRP values were available to the treating clinicians and to the physicians responsible for infection adjudication according to the LMCI criteria. However, CRP is not part of the LMCI criteria used for infection classification.

Although some infection-related data were obtained within 24 hours after admission, these variables most likely reflect an infectious process already present at ICU entry. Given that infection rarely progresses from absence to fulminant sepsis within such a short timeframe, this strategy preserves diagnostic relevance.

Shock was defined as a cardiovascular SOFA score of 3 or more at ICU admission, equal to using a vasopressor (norepinephrine or epinephrine), combined with a lactate level of >2 mmol/L.

Neutrophil count was analysed based on clinical indication and was not routinely analysed in all patients. Neutropenia was defined as a neutrophil count of $<0.5\times 10^{9}$. Leukopenia was defined as a White Blood Cell count (WBC) $<3.5\times 10^{9}$. Body temperature was obtained from the SAPS-3 score and was the highest recorded within ±1 h of ICU admission. Hypothermia was defined as a body temperature <36.0 ◦C.

The Glomerular Filtration Rate (GFR) was estimated using the Chronic Kidney Disease Epidemiology Collaboration Equation (CKD-EPI) [36]. Chronic kidney disease (CKD) was defined as a pre-admission Estimated Glomerular Filtration Rate (eGFR) <60 mL/min/1.73 m^2^, calculated from the most recent creatinine measurement obtained 7–365 days before ICU admission. NGAL models were adjusted for CKD as a binary covariate, given that CKD may elevate NGAL independently of sepsis. Acute kidney injury was defined as an increase in creatinine at admission of more than or equal to 1.5 times baseline creatinine.

Microbiological cultures were defined as clinically relevant if there was growth or detection of a pathogen from a culture taken within ±48 hours of ICU admission, regardless of the anatomical site of culturing. The following culture results were considered clinically irrelevant [37]:

- Yeast fungi from non-sterile anatomical sites (e.g., airways, lower urinary tract, skin lesions)
- Potentially colonising bacteria of the upper or lower respiratory tract, if found in only one airway culture: Moraxella sp., coagulase-negative Staphylococci, viridans (alpha hemolytic) Streptococci
- Potential skin contaminants found in only one blood culture: coagulase-negative Staphylococci, viridans (alpha hemolytic) Streptococci, micrococcus sp., Propionibacterium Acnes, Corynebacterium sp., Bacillus sp.
- Unspecific culture results (e.g., “gram-positive mixed flora”, “vaginal flora”, “skin flora”, “anaerobic mixed flora”) from non-sterile anatomical sites
- Bacterial growth of Clostridium difficile, without detection of toxin
- Pneumococcus antigen tests from urinary samples

Key definitions, adjudication procedures, and time windows are summarised in Supplementary S1 Table.

### Biobank: Blood sampling, handling and storage

Upon ICU admission, biobank blood samples were collected from patients using Ethylenediaminetetraacetic acid (EDTA) treated test tubes. The samples were then centrifuged in the local hospital laboratory, aliquoted, and subsequently frozen at −80°C. If there was a delay in the freezing process, the samples were kept refrigerated to maintain integrity. Ultimately, the blood samples were stored at the Swecrit biobank in Region Skåne, Lund, Sweden.

Patients were excluded from the study if: 1) samples were either not collected or were incorrectly labelled, 2) patients were transferred between participating ICUs without new blood samples being drawn, and 3) the patient decided to withdraw from the study.

Regarding NGAL kinetics, additional samples were excluded based on specific sample handling criteria as outlined by Pedersen et al. (2010) [38]:

- Samples stored at room temperature for more than 72 hours post-collection
- Samples stored in a refrigerator for more than 7 days post-collection
- Samples exhibiting hemolysis

After the collection period, all samples were sent to the Department of Clinical Chemistry, Uppsala University Hospital, Uppsala, Sweden, where they were thawed and analysed.

Enzyme-linked immunosorbent assay (ELISA) analyses of NGAL were carried out using a commercial sandwich immunoassay kit (DY1757, R&D Systems, Minneapolis, MN, USA). A monoclonal antibody specific for human NGAL was coated onto microtiter plates. Samples and standards were pipetted into the wells and incubated for 2 h at room temperature to allow NGAL to bind to the immobilised antibodies. After washing, a biotinylated NGAL-specific antibody was added and incubated for 2 h at room temperature. Following a further washing step, a streptavidin–HRP conjugate was added, and the plates were incubated for 30 min. After a final washing step, substrate solution was added, and the enzymatic reaction was stopped by lowering the pH. Absorbance was measured using a SpectraMax 250 microplate reader (Molecular Devices, Sunnyvale, CA, USA).

Concentrations were determined by comparing the optical density of the samples with the standard curve from the same plate. All assays were calibrated against highly purified recombinant human NGAL. The assay primarily detects the monomeric and dimeric forms of NGAL and exhibits no cross-reactivity with MMP-9. All measurements were performed in a blinded fashion, without knowledge of clinical data.

The analytical measuring range of the ELISA was 78.1–5,000 pg/mL (i.e., 0.078–5.0 ng/mL in the assay well). Reported patient concentrations (ng/mL) were obtained by multiplying the back-calculated well concentration by the sample dilution factor (a basic 1:50 dilution, with additional dilutions applied as needed for high concentrations).

The intra-assay variation was 4% and the total variation 6%. We avoid the term “inter-assay variation” as it is sometimes used inconsistently to denote either the total coefficient of variation or only the between-assay component. We therefore use the terms “intra-assay variation” and “total variation”, following the relation: intra-assay variation^2^ + inter-assay variation^2^ = total coefficient of variation^2^.

CRP was analysed using a Particle Enhanced Turbidimetric Assay (PETIA) with reagents from Abbott Laboratories (reagent 6K26-41 and calibrator 6K26-10; Abbott Park, IL, USA) on a Mindray BS380/BS430 chemistry analyser (Mindray, Shenzhen, China). This high-sensitivity assay can detect CRP levels as low as 0.5 mg/L. The following settings were used: R1 90 μ L, R2 60 μ L, sample 4 μ L, positive kinetic reaction type, reaction time positions 24–34. Results were reported with two decimal places. The total coefficient of variation (CV) was 1% (within-run CV 0.8%) at 20 mg/L and 1% (within-run CV 0.6%) at 73 mg/L.

All biomarker analyses were performed by investigators who were blinded to the clinical data.

### Statistical methods

All statistical analyses were performed using R version 4.4.2 [39]. P-values < 0.05 were considered statistically significant.

Median values and Interquartile Range (IQR) were reported for continuous variables. The mean value and Standard Deviation (SD) were reported for SOFA scores. The Mann-Whitney U test was used to assess the difference between non-sepsis and sepsis in independent continuous variables. Differences in proportions were evaluated using Pearson’s $\chi^{2}$ test.

Associations between NGAL, CRP, and sepsis were analysed using generalised linear models. The discriminatory ability of the biomarkers to distinguish sepsis from non-sepsis was assessed by Receiver Operating Characteristics (ROC) curve analysis, using the pROC package [40]. A bootstrap test with 1,000 replicates was used to compare AUC values.

**Fig 1:**
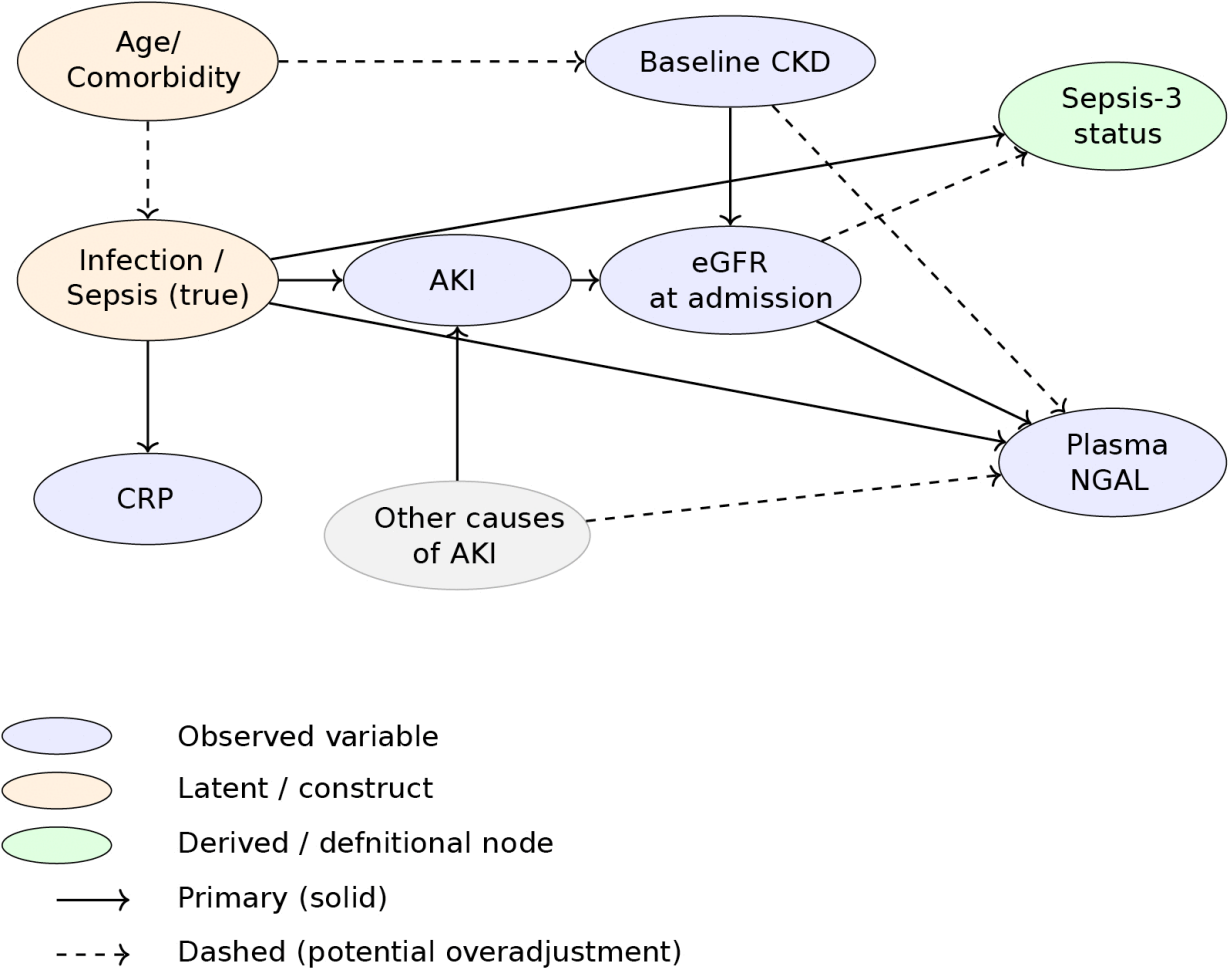
Directed acyclic graph (DAG) illustrating assumed causal relationships between infection, kidney function, NGAL, and sepsis classification.

A Directed Acyclic Graph (DAG) was constructed to map presumed causal relations between sepsis, AKI, CKD, and plasma NGAL, see Fig 1. The DAG includes potential noninfectious causes of AKI that may also affect NGAL. Based on the DAG, we adjusted primary models for baseline CKD (a potential confounder). Still, we did not adjust for AKI or concurrent eGFR because these variables lie on the causal pathway from sepsis to NGAL, and adjustment would constitute overadjustment.

We report stratified results by kidney function subgroup (No kidney injury; AKI only; CKD only; CKD and AKI) to describe diagnostic performance across clinical phenotypes, noting that stratification conditions on AKI may induce selection/collider bias.

Likelihood ratio tests were conducted to evaluate AKI, CKD and age as interaction terms.

NGAL was analysed as a continuous variable without applying a predefined test positivity threshold.

For the regression analyses, NGAL was log10-transformed and z-normalised, and CRP was fourth-root transformed and z-normalised. Age was also z-normalised. These transformations were applied to mitigate data skewness and facilitate a more straightforward comparison of results.

Spearman’s rank correlation was applied for correlation analyses. To illustrate the correlation between NGAL, CRP and sepsis, Locally Estimated Scatterplot Smoothing (LOESS) regression was calculated using the ggplot2 package [41].

To complement hypothesis testing in this large cohort, we quantified incremental value using ΔAUC with bootstrap 95% confidence intervals. We additionally evaluated clinical utility using decision-curve analysis (net benefit across plausible threshold probabilities) and reported sensitivity, specificity, positive predictive value (PPV), and negative predictive value (NPV) at pre-specified, clinically plausible cut points. Exploratory operating characteristics were calculated at pre-specified NGAL thresholds of 100, 200, 400 and 600 ng/mL. These thresholds were selected to represent clinically plausible biomarker ranges and correspond approximately to the lower quartile, median, upper quartile and high NGAL concentrations observed in the study population.

A sensitivity analysis was performed in which patients were stratified according to kidney function status. The cohort was divided into four groups: (1) CKD with AKI, patients with pre-existing CKD who developed AKI at ICU admission; (2) CKD only, patients with CKD but no AKI; (3) AKI only, patients without CKD who had developed AKI at ICU admission; and (4) no kidney injury, patients without CKD or AKI. In an additional sensitivity analysis, we recalculated the SOFA score, excluding the renal component and assessed how many patients originally classified as having sepsis no longer fulfilled the SOFA ≥2 criterion.

### Bias

CRP levels were available to clinicians in the ICU, which could contribute to the diagnosis of sepsis. This might introduce a bias, resulting in a higher AUC for CRP as a diagnostic marker for sepsis. To reduce confirmation bias in evaluating the diagnostic capability of CRP, we implemented infection criteria that include all culture-positive patients. For culture-negative sepsis, we used blood culture sampling and antibiotic administration as proxy criteria, combined with assessments according to the LMCI.

Our research group previously conducted a study on the same population, with a dropout analysis performed to determine whether missing biobank samples occurred at random.

Inclusion required an ICU stay > 24 hours (or death within 24 hours), which may have enriched the cohort for more severely ill patients. The direction of this spectrum bias is relevant for interpreting NGAL’s diagnostic performance. Patients with prolonged ICU stays are more likely to have sustained acute kidney injury, and AKI raises NGAL concentrations independently of infection. In an AKI-enriched cohort, NGAL is therefore elevated in a substantial proportion of non-septic patients, attenuating its specificity for sepsis and likely contributing to the modest AUC observed. This is consistent with the dropout analysis, in which NGAL levels did not differ between included and excluded patients despite the included cohort having a higher prevalence of sepsis, suggesting that AKI-related NGAL elevation may partially obscure the infection-specific signal.

### Missing data

No data for plasma NGAL, CRP, creatinine at ICU admission, pre-admission creatinine (7–365 days before ICU admission), age, or sex were missing.

Missing data were minimal for leukocyte count, with 16 values missing (0.5%). In contrast, neutrophil counts were missing in 2,890 patients (98%), as this test was not part of routine ICU admission laboratory testing and was typically only performed when a hematological disorder was suspected.

## Results

### Participants

**Fig 2:**
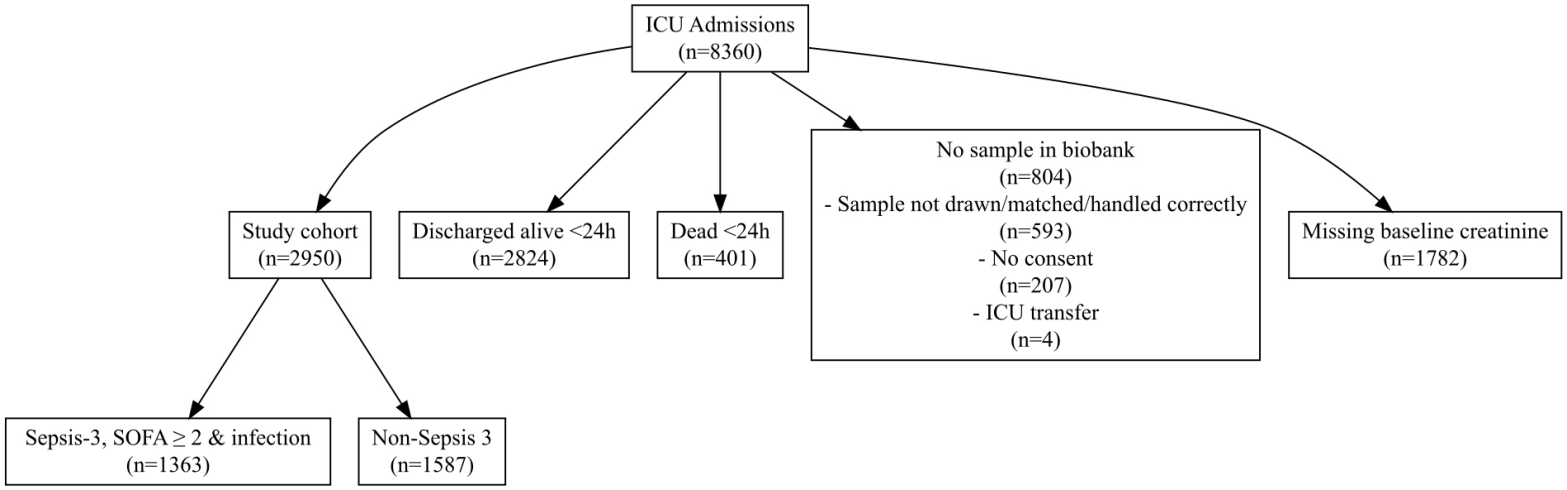
Flow chart of patient inclusion and exclusion. The included cohort had an ICU length of stay of >24 hours or died ≤24 hours after ICU admission and had eligible biobank blood samples. Of these, 46% had sepsis. Infection criteria were culture-positive ± 48 hours or blood culture & antibiotics & probable infection according to LMCI. ICU, intensive care unit; LMCI, Linder–Mellhammar criteria of infection.

During the study period, a total of 8360 ICU patients were assessed, of which 2950 met the inclusion criteria. Among these, 1363 patients (46%) satisfied the criteria for sepsis. Notably, 81% of the sepsis cases were identified based on positive culture results, while the remaining 19% met clinical criteria for infection despite negative cultures. For a detailed overview, see Fig 2.

### Descriptive data

Of the 2,950 included patients, 1,363 (46%) fulfilled Sepsis-3 criteria. Patients with sepsis had higher illness severity scores at ICU admission, including higher SOFA and SAPS-3 scores, and were more likely to present with shock and elevated inflammatory biomarkers compared with patients without sepsis. Patients with sepsis also had higher lactate levels and a higher proportion requiring invasive ventilation and renal replacement therapy. ICU- and 30-day mortality was higher in patients with sepsis.

Plasma NGAL levels were significantly higher in patients with sepsis compared to those without (median 304 ng/mL, IQR 150–638 ng/mL vs. 177 ng/mL, IQR 107–313 ng/mL), as were CRP levels (median 93 mg/L, IQR 29–175 mg/L vs. 22 mg/L, IQR 4–76 mg/L), see Table 1.

**Table 1:** Descriptive characteristics and clinical outcomes of patients with and without sepsis.

n	Non-sepsis	Sepsis	P-value
	1587	1363	
Age, years (median [IQR])	68.0 [57.0, 75.0]	69.0 [60.0, 76.0]	0.005
Male sex (%)	948 (59.7)	809 (59.4)	0.86
**Before ICU admission**			
ICU admission < 24h from hospital admission (%)	824 (51.9)	686 (50.3)	0.41
ICU admission > 48h from hospital admission (%)	505 (31.8)	468 (34.3)	0.16
Time to ICU, days (median [IQR])	0.0 [0.0 - 4.0]	0.0 [0.0 - 4.0]	0.108
Admitted from ER (%)	535 (34)	452 (33)	0.78
Admitted from hospital ward (%)	499 (31)	644 (47)	<0.001
Admitted from OR (%)	380 (24)	119 (8.7)	<0.001
**Biomarkers at ICU admission**			
NGAL, ng/mL (median [IQR])	177 [107-313]	304 [150-638]	<0.001
C-reactive protein, mg/L (median [IQR])	22 [4-76]	93 [29-175]	<0.001
Lactate, mmol/L (median [IQR])	1.8 [1.1-3.4]	1.9 [1.1-4.0]	0.003
WBC, 10^9^/L (median [IQR])	12.3 [8.6-16.7]	13.0 [8.0-19.0]	0.079
Leukopenia (%)	78 (4.9)	181 (13.3)	<0.001
Neutropenia (%)	11 (0.7)	49 (3.6)	<0.001
**Physiology at ICU admission**			
Shock (%)	408 (25.9)	451 (33.2)	<0.001
Body temperature, °C (median [IQR])	37.0 [36.2-37.4]	37.1 [36.4-38.0]	<0.001
Body temperature >38°C (%)	144 (9.1)	297 (21.9)	<0.001
Hypothermia (%)	231 (14.6)	199 (14.7)	1.000
Respiratory SOFA (mean (SD))	1.9 (1.3)	2.6 (1.2)	<0.001
Coagulation SOFA (mean (SD))	0.3 (0.7)	0.5 (0.9)	<0.001
Liver SOFA (mean (SD))	0.3 (0.7)	0.4 (0.8)	0.001
Cardiovascular SOFA (mean (SD))	1.9 (1.7)	2.4 (1.7)	<0.001
CNS SOFA (mean (SD))	1.1 (1.5)	1.4 (1.4)	<0.001
Renal SOFA (mean (SD))	1.0 (1.5)	1.4 (1.6)	<0.001
**SAPS-3 and SOFA score at ICU admission**			
SAPS-3 score (median [IQR])	59 [48-70]	68 [59-78]	<0.001
SOFA score (mean (SD))	6.0 (3.8)	8.2 (3.4)	<0.001
**Kidney injury group**			
No Kidney Injury (%)	903 (57)	623 (46)	<0.001
AKI only (%)	267 (17)	361 (27)	<0.001
CKD only (%)	337 (21)	261 (19)	0.174
CKD and AKI (%)	80 (5)	118 (8.7)	<0.001
**Outcomes**			
ICU LOS, days (median [IQR])	2.1 [1.4-3.8]	3.5 [1.9-6.6]	<0.001
ICU mortality (%)	186 (12)	236 (17)	<0.001
30-day mortality (%)	353 (22)	431 (32)	<0.001
CRRT (%)	186 (12)	257 (19)	<0.001
Invasive ventilation (%)	893 (56)	909 (67)	<0.001

ICU, intensive care unit; LOS, length of stay; ER, emergency room; OR, operating room; IQR, interquartile range; NGAL, neutrophil gelatinase-associated lipocalin; CRP, C-reactive protein; WBC, white blood cell count; SOFA, Sequential Organ Failure Assessment; CRRT, continuous renal replacement therapy.

As shown in (S1 Fig), higher NGAL and CRP concentrations were generally associated with a higher probability of sepsis.

NGAL and CRP were positively correlated (ρ = 0.39, p < 0.001), see (S2 Fig).

We could not reliably extract the timing of antibiotic administration relative to sampling; therefore, timing heterogeneity is discussed as an important limitation likely to dilute diagnostic accuracy estimates.

### Unadjusted and adjusted linear models

In a Generalised Linear Model adjusted for CKD and age, NGAL was associated with sepsis with an odds ratio (OR) of 1.77 (95% CI 1.64–1.92, p < 0.001). The model demonstrated modest discriminative ability with an AUC of 0.65 (95% CI 0.63–0.67).

**Fig 3:**
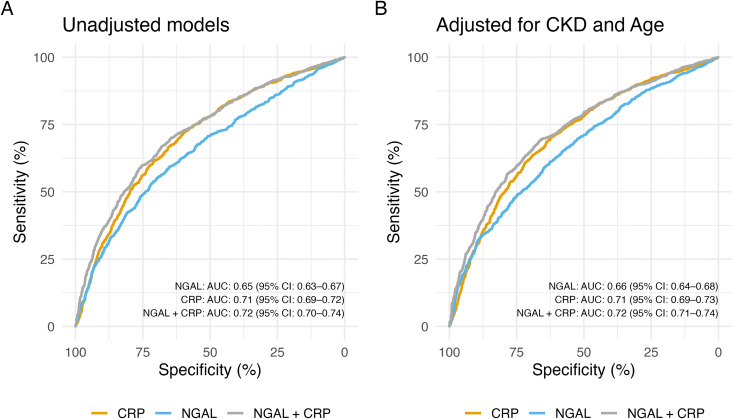
Receiver operating characteristic analysis for NGAL and CRP. (A) Receiver operating characteristic (ROC) curves and corresponding areas under the curve (AUCs) with 95% CIs for NGAL, CRP, and their combination as predictors of sepsis. (B) ROC AUCs with 95% CIs for NGAL, CRP, and their combination adjusted for chronic kidney disease and age. NGAL, neutrophil gelatinase–associated lipocalin; CRP, C-reactive protein; AUC, area under the curve; CI, confidence interval; CKD, chronic kidney disease.

**Fig 4:**
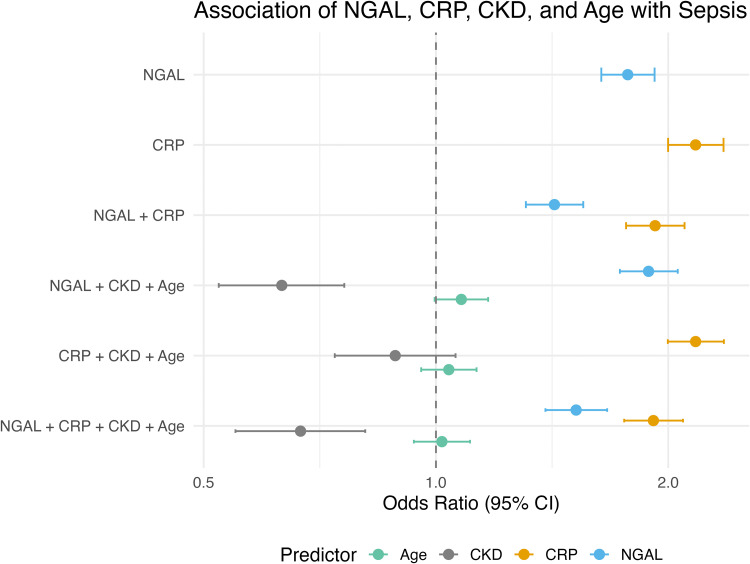
Odds ratios for sepsis associated with NGAL and CRP. Forest plot showing odds ratios (ORs) with 95% CIs for NGAL and CRP as predictors of sepsis based on generalised linear models. The models include: (1) NGAL alone; (2) CRP alone; (3) NGAL and CRP; (4) NGAL adjusted for chronic kidney disease and age; (5) CRP adjusted for chronic kidney disease and age; and (6) NGAL and CRP adjusted for chronic kidney disease and age. NGAL, neutrophil gelatinase-associated lipocalin; CRP, C-reactive protein; OR, odds ratio; CI, confidence interval; CKD, chronic kidney disease.

CRP demonstrated superior discriminatory ability for sepsis compared to NGAL, with an AUC of 0.71 (95% CI: 0.69–0.72, p < 0.001). The combination of NGAL and CRP, analysed using GLM and ROC analysis, showed improved diagnostic performance compared to CRP alone, with an AUC of 0.72 versus 0.71 (p < 0.001), ΔAUC 0.018 (95% CI: 0.011–0.027). Adjustment for CKD and age had minimal impact on the estimates (Fig 3 and Fig 4).

The discriminative ability of NGAL for sepsis remained essentially unchanged after adjustment for CKD and age. Similarly, combining NGAL with CRP yielded comparable diagnostic performance regardless of these adjustments. In the adjusted models, CKD was associated with an apparently protective odds ratio for sepsis (OR < 1.0), with wide confidence intervals (Fig 4). This is a conditional association and should not be interpreted as CKD reducing sepsis risk. Rather, it reflects that CKD patients have chronically elevated NGAL and CRP at baseline due to sustained renal inflammation and reduced clearance, independently of infection. Conditional on a given NGAL or CRP level, a patient with CKD is therefore less likely to have an infection-specific cause for that elevation than a patient without CKD. This attenuation of the infection-specific signal fraction is the likely explanation for the apparent protective direction of the CKD coefficient in adjusted models, and is consistent with the biological rationale for including CKD as a covariate.

Likelihood ratio tests (LRT) confirmed that including AKI, CKD and age as interaction terms did not significantly improve model fit compared to using them as adjustment variables (AKI p = 0.23, CKD p = 0.80, age p = 0.94).

Decision curve analysis showed largely overlapping curves for the CRP model and the CRP + NGAL model across the evaluated threshold probabilities, indicating minimal incremental clinical utility of adding NGAL (S3 Fig). Exploratory operating characteristics of NGAL across pre-specified cutoffs are presented in Supplementary S2 Table. PPV increased, and NPV decreased with increasing assumed sepsis prevalence. At a cutoff of 200 ng/mL, PPV increased from 0.14 at 10% prevalence to 0.50 at 40%, while NPV decreased from 0.94 to 0.71.

### Sensitivity analyses

**Fig 5:**
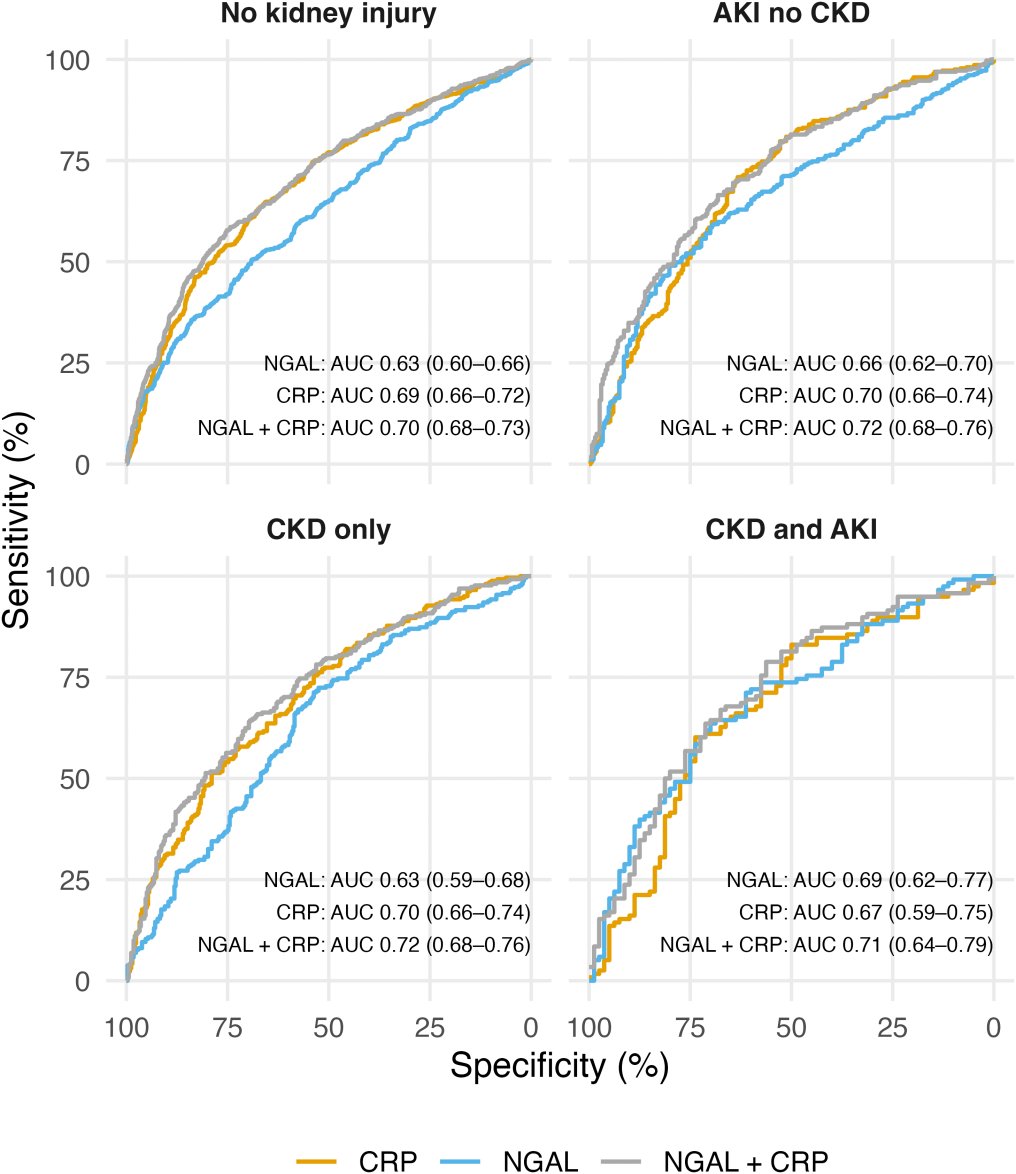
Receiver operating characteristic analysis stratified by kidney function. Receiver operating characteristic (ROC) curves and corresponding areas under the curve (AUCs) with 95% CIs for NGAL, CRP, and their combination for sepsis prediction across kidney function subgroups. NGAL, neutrophil gelatinase-associated lipocalin; CRP, C-reactive protein; AUC, area under the curve; CI, confidence interval.

We evaluated whether adding NGAL to CRP improved discrimination for sepsis across strata defined by kidney-function phenotype (no kidney injury; AKI only; CKD only; CKD and AKI). As shown in Fig 5, overall discrimination differed across strata. In these stratified (descriptive) analyses, adding NGAL to CRP was associated with only small changes in AUC in some strata (notably the “no kidney injury” and “AKI only” groups). Because stratification conditions on AKI status and may introduce collider/selection bias, these subgroup findings are presented as exploratory and should not be interpreted causally. In the CKD-only and CKD + AKI strata, the AUC differences did not reach statistical significance (p = 0.062 and p = 0.083, respectively). Notably, in the CKD and AKI stratum, NGAL numerically exceeded CRP (AUC 0.69 vs 0.67), the only subgroup showing this pattern; this likely reflects the magnitude of acute-on-chronic renal injury rather than a specific infection signal, and the wide confidence intervals preclude any firm conclusion.

In a sensitivity analysis excluding the renal SOFA component, 23 of 1,363 patients (1.7%) originally classified as having sepsis no longer fulfilled the Sepsis-3 criterion of ≥2.

### Dropout analysis

Of 5,536 eligible patients, 2,950 were included and 2,586 were excluded. Excluded patients were younger and more often admitted from the emergency room, whereas included patients more frequently had longer hospital stays prior to ICU admission (all p < 0.001).

CRP was higher in included patients, while lactate and WBC were higher in excluded patients; NGAL levels did not differ between groups. Sepsis was more common among included patients, whereas shock was more frequent in excluded patients.

Despite similar illness severity scores (SOFA and SAPS-3), excluded patients had higher ICU and 30-day mortality. See Supplementary S3 Table.

## Discussion

In this large multicentre ICU cohort, plasma NGAL measured at ICU admission demonstrated modest discrimination for Sepsis-3, with an AUC of 0.66. CRP showed superior performance (AUC 0.71), and adding NGAL to CRP resulted in a small increase in AUC (0.72 vs 0.71). Although statistically significant in this large sample, the absolute improvement in discrimination was limited. Decision-analytic and operating-characteristic analyses likewise suggested that the incremental value of NGAL over CRP at ICU admission is small in clinical terms.

Because kidney dysfunction substantially influences NGAL concentrations, we carefully addressed baseline kidney function and acute kidney injury in both primary and sensitivity analyses. Baseline CKD and age were treated as potential confounders, as both may affect baseline NGAL concentrations and susceptibility to infection. In contrast, AKI may lie on the causal pathway from infection to NGAL elevation. Adjusting for AKI could therefore attenuate the total diagnostic signal and introduce overadjustment or collider bias. Accordingly, primary models adjusted for CKD and age but not for AKI. Discriminatory performance remained largely unchanged after adjustment, and results were robust across alternative baseline-creatinine handling strategies. Nevertheless, given the close biological and clinical coupling between sepsis and AKI, complete separation of infection-related and kidney-related signals is not possible.

Research on NGAL as a diagnostic marker for sepsis remains limited [19,21–25]. Much of the literature has focused instead on NGAL as a predictor of sepsis-associated AKI, where evidence is stronger [15,16]. Sepsis and AKI are closely intertwined pathophysiologically; AKI occurs in a substantial proportion of septic ICU patients [10], and sepsis is a leading cause of AKI in hospitalised populations [42]. NGAL, as an acute-phase protein involved in innate immune responses and tubular stress, is therefore biologically plausible as a sepsis biomarker. However, the same biological properties that make NGAL sensitive to infection also render it sensitive to non-infectious kidney injury and systemic inflammation, reducing specificity for sepsis in heterogeneous ICU populations.

Compared with prior smaller studies reporting higher AUCs for NGAL in sepsis [19,21], our results are more modest. Differences in patient spectrum, reference standards, timing of sampling, and handling of kidney function likely contribute to these discrepancies. Our cohort included a broad ICU case-mix with substantial renal dysfunction and timing heterogeneity relative to symptom onset and treatment, factors that may attenuate apparent diagnostic performance. In addition, CRP was available to clinicians as part of routine care, which may have influenced diagnostic workup and documentation; although we used predefined infection criteria to mitigate incorporation bias, some residual bias cannot be excluded.

The observed improvement when adding NGAL to CRP, while statistically detectable, was small in magnitude. In a setting where CRP is already routinely available and inexpensive, the addition of NGAL at ICU admission would need to meaningfully alter clinical decision-making to justify implementation. Our analyses do not suggest such a clinically relevant gain in this population.

### Strengths and limitations

The principal strength of this study is its large, multicentre design, with prospectively collected biobank samples obtained at ICU admission, which enhances precision and external validity in similar ICU settings. Infection adjudication for culture-negative cases was performed using a structured LMCI-based review process, and NGAL measurements were conducted blinded to clinical data. The use of multiple sensitivity analyses addressing baseline kidney function and the renal component of SOFA strengthens internal validity.

Several limitations warrant consideration. First, CRP values were available in routine care and may have influenced clinical documentation and sepsis classification, introducing potential incorporation bias despite predefined infection criteria. Second, NGAL was measured at a single time point. Biomarker performance in sepsis is time-dependent and influenced by the timing of infection onset, antibiotic administration, haemodynamic resuscitation, and evolving renal injury. Heterogeneity in these factors likely attenuates discrimination estimates. Third, stratified kidney-phenotype analyses condition on AKI status and may introduce collider or selection bias; these results should therefore be interpreted as descriptive rather than causal. Finally, this study includes only critically ill ICU patients with substantial organ dysfunction and findings may not be generalisable to patients with less severe disease, shorter ICU stays, or those not admitted to the ICU.

## Conclusion

In this large multicentre ICU cohort, NGAL measured at ICU admission showed modest discrimination for Sepsis-3. Although adding NGAL to CRP resulted in a statistically detectable improvement in model performance, the magnitude of this improvement was small and unlikely to translate into meaningful clinical benefit. Our findings do not support the routine use of NGAL for sepsis diagnosis at ICU admission.

## Supporting information

S1 Fig The probability of sepsis in relation to NGAL and CRP. The solid line (LOESS) shows the probability of sepsis (left y-axis) in relation to NGAL and CRP. Points indicate sepsis or no sepsis (right y-axis). NGAL, neutrophil gelatinase–associated lipocalin; CRP, C-reactive protein.
(TIF)

S2 Fig Relationship between NGAL and CRP. Scatterplot of NGAL and CRP, with NGAL on log10 scale and CRP on fourth-root scale. Solid line is local polynomial regression fit. NGAL, neutrophil gelatinase–associated lipocalin; CRP, C-reactive protein.
(TIF)

S3 Fig Decision curve analysis of CRP and CRP + NGAL for sepsis diagnosis. Net benefit across threshold probabilities comparing a model including CRP with a model including CRP and NGAL. NGAL, neutrophil gelatinase–associated lipocalin; CRP, C-reactive protein.
(TIF)

S1 Table Definitions and adjudication procedures. Summary of key study definitions, infection adjudication procedures, time windows, and blinding. LMCI, Linder-Mellhammar Criteria of Infection; SOFA, Sequential Organ Failure Assessment; NGAL, neutrophil gelatinase–associated lipocalin; CRP, C-reactive protein.
(PDF)

S2 Table Operating characteristics of NGAL for sepsis diagnosis. Values are shown at four NGAL thresholds (100, 200, 400, 600 ng/mL). TP = true positives; FP = false positives; FN = false negatives; TN = true negatives; Sensitivity = TP / (TP + FN); Specificity = TN / (TN + FP); PPV = positive predictive value; NPV = negative predictive value; PPV (x%) and NPV (x%) denote predictive values assuming a sepsis prevalence of x%.
(PDF)

S3 Table Baseline characteristics of included and excluded patients. Excluded patients lacked biobank blood samples or baseline creatinine measurements. All variables, except outcomes, were assessed at ICU admission. CRRT and invasive mechanical ventilation refer to treatments administered during the ICU stay. ICU, intensive care unit; LOS, length of stay; ER, emergency room; IQR, interquartile range; SD, standard deviation; CRP, C-reactive protein; WBC, white blood cell count; SAPS-3, Simplified Acute Physiology Score 3; SOFA, Sequential Organ Failure Assessment; CRRT, continuous renal replacement therapy.
(PDF)

S1 Checklist Completed STARD checklist for the study (provided as a separate file). (PDF)
