# Supplementary material for: Neutrophil gelatinase-associated lipocalin (NGAL) is a poor diagnostic marker for sepsis in the ICU – An observational multicentre study
Source: PLoS One. 2026 Aug 14;21(8):e0343752. doi: 10.1371/journal.pone.0343752 (PMC13475940; doi:10.1371/journal.pone.0343752)
Supplement: Supplementary file 1 [file pone.0343752.s004.pdf]

S1 Table. Definitions and adjudication procedures.

Domain	Definition
**Infection definition**	(1) Culture positivity within ±48 h of ICU admission; or (2) Culture-negative suspected infection (blood cultures within 24 h and antibiotic treatment) with probable infection according to LMCI criteria.
**Infection adjudication**	Retrospective adjudication by five physicians (three residents, two specialists) using a structured LMCI-based protocol. Reviewers were trained by a single instructor; uncertain cases were resolved by consensus. Interrater variability was not formally assessed.
**Sepsis**	SOFA score $\geq$2 within ±1 h of ICU admission plus retrospectively adjudicated infection (Sepsis-3).
**Shock**	Cardiovascular SOFA $\geq$3 (vasopressor use) and lactate $>$2 mmol/L at ICU admission.
**Biomarker window**	Values closest to ICU admission within 24 h were used.
**Infection window**	Microbiological cultures and infection classification based on data within ±48 h of ICU admission.
**Blinding**	NGAL values were not available to clinicians or adjudicators. CRP was available but not part of LMCI criteria.

Summary of key study definitions and adjudication procedures. LMCI, Linder-Mellhammar Criteria of Infection; SOFA, Sequential Organ Failure Assessment; NGAL, neutrophil gelatinase–associated lipocalin; CRP, C-reactive protein.
