# Supplementary material for: Neutrophil gelatinase-associated lipocalin (NGAL) is a poor diagnostic marker for sepsis in the ICU – An observational multicentre study
Source: PLoS One. 2026 Aug 14;21(8):e0343752. doi: 10.1371/journal.pone.0343752 (PMC13475940; doi:10.1371/journal.pone.0343752)
Supplement: Supplementary file 2 [file pone.0343752.s005.pdf]

S2 Table. Diagnostic performance of plasma NGAL at predefined cutoffs for prediction of sepsis.

	**100 ng/mL**	**200 ng/mL**	**400 ng/mL**	**600 ng/mL**
TP	1201	884	532	369
FP	1237	686	278	157
FN	162	479	831	994
TN	350	901	1309	1430
Sensitivity	0.88	0.65	0.39	0.27
Specificity	0.22	0.57	0.82	0.90
PPV (10%)	0.11	0.14	0.20	0.23
NPV (10%)	0.94	0.94	0.92	0.92
PPV (20%)	0.22	0.27	0.36	0.41
NPV (20%)	0.88	0.87	0.84	0.83
PPV (30%)	0.33	0.39	0.49	0.54
NPV (30%)	0.81	0.79	0.76	0.74
PPV (40%)	0.43	0.50	0.60	0.65
NPV (40%)	0.74	0.71	0.67	0.65

TP, true positives; FP, false positives; FN, false negatives; TN, true negatives; PPV, positive predictive value; NPV, negative predictive value.
