# Supplementary material for: Neutrophil gelatinase-associated lipocalin (NGAL) is a poor diagnostic marker for sepsis in the ICU – An observational multicentre study
Source: PLoS One. 2026 Aug 14;21(8):e0343752. doi: 10.1371/journal.pone.0343752 (PMC13475940; doi:10.1371/journal.pone.0343752)
Supplement: Supplementary file 3 [file pone.0343752.s006.pdf]

S3 Table. Baseline characteristics of included and excluded patients.

	Included (n=2950)	Excluded (n=2586)	p-value
**Demographics**			
Age, years	69 [59-75]	66 [52-75]	<0.001
Male sex, n (%)	1757 (60)	1566 (61)	0.466
ICU admission >48h from hospital admission, n (%)	973 (33)	487 (19)	<0.001
Admitted from ER, n (%)	987 (34)	1263 (49)	<0.001
**Biomarkers**			
NGAL, ng/mL	217 [122-439]	207 [113-447]	0.136
C-reactive protein, mg/L	47 [9-127]	28 [3-128]	<0.001
Lactate, mmol/L	1.9 [1.1-3.7]	2.2 [1.2-4.7]	<0.001
WBC, 10^9/L	12.6 [8.3-17.6]	13.3 [9.3-18.5]	<0.001
**Physiological parameters**			
Sepsis, n (%)	1363 (46)	968 (37)	<0.001
Shock, n (%)	859 (29)	868 (34)	<0.001
SOFA score (mean (SD))	7.0 (3.8)	7.1 (4.3)	0.859
SAPS-3 score (median [IQR])	63 [53-74]	63 [50-75]	0.206
**Outcomes**			
ICU LOS, days (median [IQR])	2.6 [1.7-5.0]	2.2 [1.2-4.6]	<0.001
ICU mortality, n (%)	422 (14)	624 (24)	<0.001
30-day mortality, n (%)	784 (27)	903 (36)	<0.001
CRRT, n (%)	443 (15)	271 (11)	<0.001
Invasive mechanical ventilation, n (%)	1802 (61)	1706 (66)	<0.001

Excluded patients lacked biobank blood samples or baseline creatinine measurements. All variables, except outcomes, were assessed at ICU admission. CRRT and invasive mechanical ventilation refer to treatments administered during the ICU stay. ICU, intensive care unit; LOS, length of stay; ER, emergency room; IQR, interquartile range; SD, standard deviation; CRP, C-reactive protein; WBC, white blood cell count; SAPS-3, Simplified Acute Physiology Score 3; SOFA, Sequential Organ Failure Assessment; CRRT, continuous renal replacement therapy.
