# Supplementary material for: Neutrophil gelatinase-associated lipocalin (NGAL) is a poor diagnostic marker for sepsis in the ICU – An observational multicentre study
Source: PLoS One. 2026 Aug 14;21(8):e0343752. doi: 10.1371/journal.pone.0343752 (PMC13475940; doi:10.1371/journal.pone.0343752)
Supplement: Supplementary file 5 [file pone.0343752.s009.pdf]

Response to Reviewer Comments

Manuscript: *Neutrophil gelatinase-associated lipocalin (NGAL) is a poor diagnostic marker for sepsis in the ICU – an observational multicentre study*

We thank the reviewer for the careful and constructive assessment. We have revised the manuscript substantially. All changes in the manuscript are highlighted using yellow highlighting (`\hl{}`).

Major comments

1) Reference standard and risk of incorporation/confirmation bias

Reviewer: Sepsis classification depends on retrospective infection adjudication combined with SOFA criteria. CRP was available to clinicians and could influence the diagnostic workup/documentation and downstream classification. Please clarify who adjudicated infection/LMCI, training/standardisation, blinding to CRP/NGAL, and disagreement handling.

Response: We agree that adjudication procedures and blinding are critical, especially since routine CRP values were available.

Manuscript changes: We have added a paragraph under Data sources and variables specifying that infection adjudication was performed by five physicians (three residents and two board-certified specialists) using a structured protocol based on the LMCI criteria. Reviewers were blinded to NGAL levels. Ambiguous cases were resolved by consensus. CRP is not included in the LMCI criteria. We have also added a summary table in the Supporting Information outlining definitions and adjudication procedures.

2) Kidney function definitions and handling of missing baseline creatinine

Reviewer: Missing baseline creatinine is 38%; assuming "no CKD" is likely incorrect. AKI definition in non-CKD as admission eGFR$<$60 is not KDIGO-consistent. Also consider sensitivity analysis excluding renal SOFA component.

Response: We appreciate the reviewers' comment and have implemented a more rigorous framework for kidney function. Patients without baseline creatinine are no longer assumed to have no CKD; only those with available baseline creatinine were included in the analyses, preserving a substantial study cohort (n = 2,950), while patients lacking baseline values were considered in a dropout analysis. To improve AKI classification, we revised the definition to follow the KDIGO creatinine-change criteria, as urine output data were not accessible. Because the Sepsis-3 definition incorporates renal SOFA points, we also performed a sensitivity analysis using a modified SOFA score that excludes the renal component. In this analysis, 23 of 1,363 patients (1.7%) initially classified as having sepsis no longer met the Sepsis-3 criterion of SOFA ≥ 2.

Manuscript changes: Revised kidney definitions and missing baseline handling in Data sources and variables and Statistical methods; added sensitivity analysis excluding renal SOFA component. Revised Table 1 and Results in accordance with changes in inclusion criteria.

3) Clinical utility: statistical significance vs meaningful improvement

Reviewer: With large n, small differences become statistically significant. Please add ΔAUC with CIs, decision-curve analysis/net benefit, sensitivity/specificity at plausible cut points, PPV/NPV across prevalence ranges.

Response: We agree. We now explicitly separate statistical significance from clinical relevance.

Manuscript changes: Added to Statistical Methods; (i) ΔAUC with bootstrap 95% CIs, (ii) decision-curve analysis across plausible thresholds, and (iii) exploratory operating characteristics (sensitivity, specificity, PPV, NPV) at pre-specified cut points and across relevant ICU prevalence ranges. Outputs are presented in Supporting Information and summarised in the Results/Discussion. Abstract/Discussion wording is revised to avoid overstating incremental value.

4) Timing heterogeneity and single time-point measurement

Reviewer: Please report time from hospital admission to ICU admission, antibiotic timing relative to sampling if possible, and discuss timing heterogeneity.

Response: We thank the reviewer for this important comment. We have added time from hospital admission to ICU admission to Table 1 to better provide context for disease progression. Data on antibiotic timing relative to NGAL sampling were not reliably available and could therefore not be reported. However, within the sepsis definition, patients without culture positivity were required to have received antibiotics in close temporal proximity to blood culture sampling (within 24 hours of ICU admission). This is clarified in the Methods section. We now explicitly acknowledge this limitation and discuss how heterogeneity in timing—both in relation to infection onset, resuscitation, and renal injury—may have influenced the observed diagnostic performance.

Manuscript changes: Added timing heterogeneity description in Results and expanded limitations in Discussion.

5) Selection/spectrum bias and generalisability

Reviewer: Inclusion requires ICU stay > 24 h (or death < 24 h) plus biobank samples, enriching for sicker patients. Strengthen description of excluded patients and generalisability statements.

Response: We agree and expanded the spectrum-bias/generalisation discussion. We also expanded the description of excluded patients using the prior dropout analysis.

Manuscript changes: Expanded Bias and Strengths and limitations sections, adding stronger generalisability caveats.

6) Subgroup analyses and collider/selection bias

Reviewer: Stratifying on AKI may induce collider bias; tone down subgroup conclusions, consider interaction terms as primary.

Response: We thank the reviewer for this important point. We have now evaluated AKI as an interaction term in regression models. These analyses did not demonstrate significant effect modification. In addition, we have strengthened the discussion of potential bias arising from conditioning on AKI, noting its dual role as a mediator and potential collider. In this context, both stratified and interaction-based analyses are interpreted cautiously and viewed as complementary approaches to explore the influence of kidney function, rather than as definitive evidence of causal effect modification.

Manuscript changes: Revised Results and Discussion language with stronger caveats and reduced emphasis on subgroup-based interpretations.

7) Data availability and reproducibility (PLOS ONE)

Reviewer: Provide concrete access mechanism and what will be shared (de-identified data, codebook, code).

Response: We have revised the Data Availability statement to provide a workable access pathway (ethical approval + agreement with Region Skåne).

Manuscript changes: Replaced Data Availability section with a concrete request process and explicit reproducibility artefacts.

Minor comments

1) Terminology duplication (NGAL expansion repeated)

Response: Corrected throughout, including the Abstract. **Manuscript changes:** Edited duplicated expansions.

2) Units consistency (pg/mL vs ng/mL)

Response: We standardised reporting and clarified how dilution factors map well to concentration to reported plasma concentrations.

Response: We have standardised the units throughout the manuscript and clarified the calculation of patient plasma concentrations from ELISA measurements.

Manuscript changes: Revised ELISA range paragraph with explicit unit conversion and dilution reporting.

3) Add a compact definition box/table (sepsis, adjudication, windows, blinding)

Response: We summarised key timing windows and blinding in text; we also reference Supporting Information for a compact summary table. **Manuscript changes:** Added Table of definitions and adjudication procedures in Supporting information and a STARD checklist note.

4) Missingness beyond baseline creatinine (WBC/neutrophils)

Response: We now report missingness proportions for WBC and neutrophils. **Manuscript changes:** Updated Missing data section.

5) Comparator context (no procalcitonin)

Response: We added an explicit statement acknowledging that PCT and other biomarkers were not available in this dataset (or not harmonised across sites) and therefore are not included as comparators. **Manuscript changes:** Added explicit limitation in Discussion.

6) STARD checklist

Response: We added a statement that a completed STARD checklist is provided as Supporting Information. **Manuscript changes:** Added STARD checklist note in Methods.

We hope the revised manuscript addresses the reviewer's concerns and improves interpretability and clinical applicability.
