# Supplementary material for: Neutrophil gelatinase-associated lipocalin (NGAL) is a poor diagnostic marker for sepsis in the ICU – An observational multicentre study
Source: PLoS One. 2026 Aug 14;21(8):e0343752. doi: 10.1371/journal.pone.0343752 (PMC13475940; doi:10.1371/journal.pone.0343752)
Supplement: Peer review history

# Editor report: Decision Letter 0

**DOI:** 10.1371/journal.pone.0343752.r001

**Authors:** Hesham Keryakos

22 Feb 2026

Dear Dr. Boström,

Thank you for submitting your manuscript to PLOS ONE. After careful consideration, we feel that it has merit but does not fully meet PLOS ONE’s publication criteria as it currently stands. Therefore, we invite you to submit a revised version of the manuscript that addresses the points raised during the review process.

Please submit your revised manuscript by Apr 08 2026 11:59PM. If you will need more time than this to complete your revisions, please reply to this message or contact the journal office at plosone@plos.org. When you're ready to submit your revision, log on to https://www.editorialmanager.com/pone/ and select the 'Submissions Needing Revision' folder to locate your manuscript file.

- A letter that responds to each point raised by the academic editor and reviewer(s). You should upload this letter as a separate file labeled 'Response to Reviewers'.
- A marked-up copy of your manuscript that highlights changes made to the original version. You should upload this as a separate file labeled 'Revised Manuscript with Track Changes'.
- An unmarked version of your revised paper without tracked changes. You should upload this as a separate file labeled 'Manuscript'.

If you would like to make changes to your financial disclosure, please include your updated statement in your cover letter. Guidelines for resubmitting your figure files are available below the reviewer comments at the end of this letter.

If applicable, we recommend that you deposit your laboratory protocols in protocols.io to enhance the reproducibility of your results. Protocols.io assigns your protocol its own identifier (DOI) so that it can be cited independently in the future. For instructions see: https://journals.plos.org/plosone/s/submission-guidelines#loc-laboratory-protocols. Additionally, PLOS ONE offers an option for publishing peer-reviewed Lab Protocol articles, which describe protocols hosted on protocols.io. Read more information on sharing protocols at https://plos.org/protocols?utm_medium=editorial-email&utm_source=authorletters&utm_campaign=protocols.

We look forward to receiving your revised manuscript.

Kind regards,

Hesham K H Keryakos, Ph.D., M.D.

Academic Editor

PLOS One

**Journal Requirements:**

When submitting your revision, we need you to address these additional requirements.

1. Please ensure that your manuscript meets PLOS ONE's style requirements, including those for file naming. The PLOS ONE style templates can be found at

https://journals.plos.org/plosone/s/file?id=wjVg/PLOSOne_formatting_sample_main_body.pdf and

https://journals.plos.org/plosone/s/file?id=ba62/PLOSOne_formatting_sample_title_authors_affiliations.pdf

2. Please provide additional details regarding participant consent. In the ethics statement in the Methods and online submission information, please ensure that you have specified (1) whether consent was informed and (2) what type you obtained (for instance, written or verbal, and if verbal, how it was documented and witnessed). If your study included minors, state whether you obtained consent from parents or guardians. If the need for consent was waived by the ethics committee, please include this information.

3. If you are reporting a retrospective study of medical records or archived samples, please ensure that you have discussed whether all data were fully anonymized before you accessed them and/or whether the IRB or ethics committee waived the requirement for informed consent. If patients provided informed written consent to have data from their medical records used in research, please include this information.

4. We note that the grant information you provided in the ‘Funding Information’ and ‘Financial Disclosure’ sections do not match.

When you resubmit, please ensure that you provide the correct grant numbers for the awards you received for your study in the ‘Funding Information’ section.

5. Please note that funding information should not appear in any section or other areas of your manuscript. We will only publish funding information present in the Funding Statement section of the online submission form. Please remove any funding-related text from the manuscript.

6. In the online submission form, you indicated that your data is available only on request from a third party. Please note that your Data Availability Statement is currently missing contact details for the third party, such as an email address or a link to where data requests can be made]. Please update your statement with the missing information.

7. Please remove your figures from within your manuscript file, leaving only the individual TIFF/EPS image files, uploaded separately. These will be automatically included in the reviewers’ PDF.

8. If the reviewer comments include a recommendation to cite specific previously published works, please review and evaluate these publications to determine whether they are relevant and should be cited. There is no requirement to cite these works unless the editor has indicated otherwise.

**Additional Editor Comments:**

Comments to the Authors

Thank you for submitting this large multicentre ICU cohort study (n=4732) evaluating admission plasma NGAL for discriminating Sepsis-3 at ICU entry, benchmarked against CRP. The sample size, multicentre setting, and standardized biobank sampling at ICU admission are clear strengths. Your main message—that NGAL alone has only modest discrimination and that adding NGAL to CRP yields only a small improvement—could be valuable to the field. However, several design/definition and reporting issues currently limit interpretability and clinical applicability. I recommend major revision.

Major comments

1) Reference standard and risk of incorporation/confirmation bias

Sepsis classification depends on retrospective infection adjudication combined with SOFA criteria. At the same time, CRP was available to clinicians, and could influence diagnostic workup/documentation and downstream classification—making the NGAL vs CRP comparison harder to interpret.

Please clarify explicitly:

• Who performed infection adjudication / LMCI assessment and what training/standardization was used.

• Whether adjudicators were blinded to CRP and other inflammatory markers, and whether they were blinded to NGAL (assay blinding is described, but adjudication blinding is the key issue here).

• How disagreements were handled (single reviewer vs dual review; adjudication rules).

If adjudicators had access to CRP, then CRP is partly embedded in the diagnostic pathway, which may inflate its apparent performance and distort incremental-value claims.

2) Kidney function definitions and handling of missing baseline creatinine (high impact)

Kidney dysfunction strongly affects NGAL. Your renal subgrouping and adjustment strategy therefore needs to be very robust.

Two issues are particularly problematic:

a) Missing baseline creatinine and CKD misclassification

You report that pre-admission creatinine (7–365 days) is missing in ~38% and you assume “no CKD” for these patients. That assumption is likely incorrect and can bias:

• CKD adjustment,

• renal subgroup analyses, and

• any conclusions about NGAL’s performance “independent” of CKD.

Suggested fixes (at minimum sensitivity analyses):

• Treat missing baseline creatinine as an “unknown baseline” category rather than “no CKD”.

• Use multiple reasonable baseline strategies and show robustness (e.g., back-calculation approaches, imputation frameworks, or restricting analyses to those with known baseline).

• Report how many patients shift CKD category under each strategy.

b) AKI definition in patients without CKD

Defining AKI as eGFR <60 at ICU admission in those without CKD is not KDIGO-consistent and conflates chronic impairment, acute changes, and non-renal factors (e.g., muscle mass, fluid balance). This can substantially distort the renal-stratified results.

Suggested fixes:

• Re-define AKI using KDIGO creatinine change (and urine output if available).

• If urine output is unavailable, at least use creatinine dynamics rather than a single eGFR threshold.

• Present renal subgroup results only after a more defensible AKI/CKD classification is implemented, or clearly label them as exploratory with strong caveats.

Additional important point: Because Sepsis-3 requires SOFA ≥2 and SOFA includes a renal component, there is a risk that NGAL (as a kidney injury marker) correlates with the outcome partly through renal SOFA rather than infection biology. Consider a sensitivity analysis defining sepsis using SOFA excluding the renal component (or adjusting/stratifying by non-renal SOFA) to test whether discrimination is driven by renal dysfunction.

3) Clinical utility: statistical significance vs meaningful improvement

With this sample size, small performance differences will be statistically significant. The AUC increase when adding NGAL to CRP appears small; the manuscript would benefit from clinical interpretation, not only p-values.

Please consider adding:

• ΔAUC with confidence intervals (not only p-values),

• decision-curve analysis / net benefit (or other decision-analytic framing),

• sensitivity/specificity at clinically plausible cut points (even if exploratory),

• PPV/NPV across relevant prevalence ranges (ICU sepsis prevalence varies).

This would help readers decide whether the incremental gain is actionable.

4) Timing heterogeneity and single time-point measurement

NGAL is measured once at/near ICU admission. In sepsis, biomarker performance depends on timing relative to symptom onset, antibiotics, resuscitation, and evolving renal insult.

If possible, please report distributions for:

• time from hospital admission to ICU admission,

• timing of antibiotics relative to sampling (if available),

• proportion admitted directly from ED vs ward/OR (you partly present this).

At minimum, expand the discussion on how timing heterogeneity likely dilutes or biases diagnostic accuracy.

5) Selection/spectrum bias and generalizability

Inclusion requires ICU stay >24h (or death ≤24h) plus eligible biobank samples. This enriches for sicker patients and may alter diagnostic performance (spectrum effects).

Please strengthen:

• the description of excluded patients (and how they differ, if data exist),

• generalizability statements (ICU-only, enriched severity; not necessarily applicable to ED/ward).

6) Subgroup analyses and collider/selection bias

You appropriately acknowledge that stratifying on AKI may induce collider bias, yet subgroup conclusions are emphasized. Please:

• tone down causal/interpretive language for renal subgroups,

• consider interaction terms in a single model as the primary approach,

• present stratified results as descriptive with stronger caveats.

7) Data availability and reproducibility (PLOS ONE requirement)

Your data availability statement indicates restrictions under Swedish law. PLOS ONE generally expects a clear, workable access pathway.

Please provide:

• a concrete access mechanism (data access committee/contact, application steps, approvals required, expected timelines),

• what will be shared (de-identified dataset? derived variables? codebook/data dictionary? analysis code),

• ideally, share analysis code and a variable dictionary even if the full dataset cannot be public.

Minor comments (clarity and presentation)

1. Terminology duplication: The abstract contains repeated parenthetical expansion of NGAL (e.g., “Neutrophil gelatinase-associated lipocalin (Neutrophil gelatinase-associated lipocalin (NGAL))”). Please correct throughout.

2. Units consistency: The NGAL assay range is presented in pg/mL, while results are shown in ng/mL. Please standardize units and explicitly describe conversions and dilution reporting.

3. Outcome definition clarity: Consider adding a compact table/box summarizing:

o sepsis definition,

o infection adjudication steps (culture-positive vs culture-negative),

o LMCI elements,

o timing windows (±1h, ±48h, etc.),

o blinding status of record reviewers.

4. Missingness reporting: Beyond baseline creatinine, please report missingness for WBC/neutrophils and how this affects comparisons.

5. Comparators/context: CRP is a reasonable comparator, but absence of other widely used ICU biomarkers (e.g., procalcitonin) limits contextualization. If unavailable, acknowledge explicitly.

6. STARD checklist: If claiming STARD adherence, ensure all key items are explicitly addressed (patient flow, timing, blinding, handling of indeterminate/missing results).

Overall recommendation

Major revision. The dataset and question are strong, but the kidney-function definitions/missing baseline creatinine handling and reference-standard bias issues must be clarified and supported by sensitivity analyses before the conclusions—particularly renal subgroup interpretations and incremental value over CRP—can be considered robust.

[Note: HTML markup is below. Please do not edit.]

[NOTE: If reviewer comments were submitted as an attachment file, they will be attached to this email and accessible via the submission site. Please log into your account, locate the manuscript record, and check for the action link "View Attachments". If this link does not appear, there are no attachment files.]

To ensure your figures meet our technical requirements, please review our figure guidelines: https://journals.plos.org/plosone/s/figures

You may also use PLOS’s free figure tool, NAAS, to help you prepare publication quality figures: https://journals.plos.org/plosone/s/figures#loc-tools-for-figure-preparation.

NAAS will assess whether your figures meet our technical requirements by comparing each figure against our figure specifications.

# Author comment: Author response to Decision Letter 1

**DOI:** 10.1371/journal.pone.0343752.r002

7 Apr 2026

We have made changes in the manuscript and figures to better meet the journal requirements. We have addressed all comments from the editor and the reviewer. A detailed point-by-point response has been provided and all corresponding changes have been made in the revised manuscript and figures.

Attachment Submitted filename: Response to reviewers.pdf

# Editor report: Decision Letter 1

**DOI:** 10.1371/journal.pone.0343752.r003

**Authors:** Hideshi Okada

8 Jul 2026

Dear Dr. Boström,

Thank you for submitting your manuscript to PLOS ONE. After careful consideration, we feel that it has merit but does not fully meet PLOS ONE’s publication criteria as it currently stands. Therefore, we invite you to submit a revised version of the manuscript that addresses the points raised during the review process.

Please submit your revised manuscript by Apr 08 2026 11:59PM. If you will need more time than this to complete your revisions, please reply to this message or contact the journal office at plosone@plos.org. When you're ready to submit your revision, log on to https://www.editorialmanager.com/pone/ and select the 'Submissions Needing Revision' folder to locate your manuscript file.

- A letter that responds to each point raised by the academic editor and reviewer(s). You should upload this letter as a separate file labeled 'Response to Reviewers'.
- A marked-up copy of your manuscript that highlights changes made to the original version. You should upload this as a separate file labeled 'Revised Manuscript with Track Changes'.
- An unmarked version of your revised paper without tracked changes. You should upload this as a separate file labeled 'Manuscript'.

If you would like to make changes to your financial disclosure, please include your updated statement in your cover letter. Guidelines for resubmitting your figure files are available below the reviewer comments at the end of this letter.

If applicable, we recommend that you deposit your laboratory protocols in protocols.io to enhance the reproducibility of your results. Protocols.io assigns your protocol its own identifier (DOI) so that it can be cited independently in the future. For instructions see: https://journals.plos.org/plosone/s/submission-guidelines#loc-laboratory-protocols. Additionally, PLOS ONE offers an option for publishing peer-reviewed Lab Protocol articles, which describe protocols hosted on protocols.io. Read more information on sharing protocols at https://plos.org/protocols?utm_medium=editorial-email&utm_source=authorletters&utm_campaign=protocols.

As the corresponding author, your ORCID iD is verified in the submission system and will appear in the published article. PLOS supports the use of ORCID, and we encourage all coauthors to register for an ORCID iD and use it as well. Please encourage your coauthors to verify their ORCID iD within the submission system before final acceptance, as unverified ORCID iDs will not appear in the published article. *Only*  the individual author can complete the verification step; PLOS staff *cannot*  verify ORCID iDs on behalf of authors.

We look forward to receiving your revised manuscript.

Kind regards,

Hesham K H Keryakos, Ph.D., M.D.

Academic Editor

PLOS One

Journal Requirements:

If the reviewer comments include a recommendation to cite specific previously published works, please review and evaluate these publications to determine whether they are relevant and should be cited. There is no requirement to cite these works unless the editor has indicated otherwise.

Additional Editor Comments (if provided):

[Note: HTML markup is below. Please do not edit.]

Reviewers' comments:

[NOTE: If reviewer comments were submitted as an attachment file, they will be attached to this email and accessible via the submission site. Please log into your account, locate the manuscript record, and check for the action link "View Attachments". If this link does not appear, there are no attachment files.]

To ensure your figures meet our technical requirements, please review our figure guidelines: https://journals.plos.org/plosone/s/figures

You may also use PLOS’s free figure tool, NAAS, to help you prepare publication quality figures: https://journals.plos.org/plosone/s/figures#loc-tools-for-figure-preparation.

NAAS will assess whether your figures meet our technical requirements by comparing each figure against our figure specifications.

# Editor report: Acceptance letter

**DOI:** 10.1371/journal.pone.0343752.r004

**Authors:** Hideshi Okada

PONE-D-26-06039R1

PLOS One

Dear Dr. Boström,

I'm pleased to inform you that your manuscript has been deemed suitable for publication in PLOS One. Congratulations! Your manuscript is now being handed over to our production team.

At this stage, our production department will prepare your paper for publication. This includes ensuring the following:

* All references, tables, and figures are properly cited

* All relevant supporting information is included in the manuscript submission,

* There are no issues that prevent the paper from being properly typeset

You will receive further instructions from the production team, including instructions on how to review your proof when it is ready. Please keep in mind that we are working through a large volume of accepted articles, so please give us a few days to review your paper and let you know the next and final steps.

Lastly, if your institution or institutions have a press office, please let them know about your upcoming paper now to help maximize its impact. If they'll be preparing press materials, please inform our press team within the next 48 hours. Your manuscript will remain under strict press embargo until 2 pm Eastern Time on the date of publication. For more information, please contact onepress@plos.org.

You will receive an invoice from PLOS for your publication fee after your manuscript has reached the completed accept phase. If you receive an email requesting payment before acceptance or for any other service, this may be a phishing scheme. Learn how to identify phishing emails and protect your accounts at https://explore.plos.org/phishing.

If we can help with anything else, please email us at customercare@plos.org.

Thank you for submitting your work to PLOS ONE and supporting open access.

Kind regards,

PLOS ONE Editorial Office Staff

on behalf of

Dr. Hideshi Okada

Academic Editor

PLOS One
